# Plane Wave Imaging through Interfaces

**DOI:** 10.3390/s21154967

**Published:** 2021-07-21

**Authors:** Guillermo Cosarinsky, Jorge Fernandez-Cruza, Jorge Camacho

**Affiliations:** 1 Ultrasound Systems and Technology Group (GSTU), Institute for Physical and Information Technologies (ITEFI), Spanish National Research Council (CSIC), c/Serrano 144, 28006 Madrid, Spain; cruza@daselsistemas.com (J.F.-C.); j.camacho@csic.es (J.C.); 2 Non Destructive Testing Department, National Atomic Energy Commission (CNEA), Av. Gral. Paz 1499, Buenos Aires B1650, Argentina; 3 Electronics Department, Escuela Politécnica, Universidad de Alcalá de Henares, Ctra. Madrid-Barcelona, Km. 33,600, 28805 Madrid, Spain; 4 DASEL SL, Avda. del Cañal 44 Nave 3, 28500 Madrid, Spain

**Keywords:** ultrasound imaging, phased array, total focusing method, plane wave imaging, non-destructive-testing

## Abstract

Plane Wave Imaging (PWI) has been recently proposed for fast ultrasound inspections in the Non-Destructive-Testing (NDT) field. By using a single (or a reduced number) of plane wave emissions and parallel beamforming in reception, frame rates of hundreds to thousands of images per second can be achieved without significant image quality losses with regard to the Total Focusing Method (TFM) or Phased Array (PA). This work addresses the problem of applying PWI in the presence of arbitrarily shaped interfaces, which is a common problem in NDT. First, the mathematical formulation for generating a plane wave inside a component of arbitrary geometry is given, and the characteristics of the resultant acoustic field are analyzed by simulation, showing plane wavefronts with non-uniform amplitude. Then, an imaging strategy is proposed, accounting for this amplitude effect. Finally, the proposed method is experimentally validated, and its application limits are discussed.

## 1. Introduction

In recent years, various methods for ultrasound imaging with array transducers were developed and studied in the Non–Destructive Testing (NDT) field. The Total Focusing Method (TFM) is considered the gold standard for producing high contrast images focused both in emission and reception [1,2,3,4]. This method requires the sequential emission with each array element, followed by the reception with all elements. This acquisition mode is called Full Matrix Capture (FMC). Another method that requires less transmissions is the Plane Wave Imaging (PWI) [5,6,7,8], achieving higher image rates. In this method, each transmission uses all array elements to produce a plane wave in a specified direction. The number of transmissions is the number of plane wave directions chosen for the inspection, which can be much less than the number of elements of the array.

In NDT, it is usually necessary to deal with beam refraction at the surface of the part being inspected. This is the case of immersion testing, and also when a wedge is used between the array and the part under test. Computing the focal laws accounting for this refraction complicates the plane wave image formation process, and several authors have addressed the associated performance and frame-rate limitations [6,7,8,9,10,11]. For the Phased Array (PA) method, the focal laws can be computed using the concept of a virtual array ([9,10,11]), which is an approximation that significantly reduces the computational cost. In TFM and PWI, an exact calculation is normally used for each pixel at reception.

In [6,7,8], the authors show how plane waves can be generated inside a test piece with a complex interface, with previously known shape. In [8], they also propose the method PWAPP (Plane Wave Adapted in Post Processing), which does not require previous knowledge of the interface. Instead, an FMC capture is used, and the interface is reconstructed from this data. In our case, we use a first capture to detect the interface, using the methods described in our previous work ([9,10,11]), and then focal laws are computed to generate plane waves inside the component.

In the present work, we analyze the acoustic field generated inside a component when using focal laws to generate plane waves inside it. We use a simple simulation that shows how plane wavefronts are created in a region bounded by the rays from the extreme elements of the array, which we call the Plane Wave Region (PWR). Although this result was previously shown in previous works [6,8], here, we analyze the amplitude variations along the plane wave. The non-uniformity of the field inside the PWR might have a detrimental effect in the quality of PWI images and should be accounted for during the inspection definition.

In this work, we propose a spatial weighting strategy based on a simple field simulation to account for this effect. Finally, it is tested with experimental data, and conclusions are obtained.

## 2. Plane Wave Generation through an Arbitrary Geometry Interface

**Figure 1:**
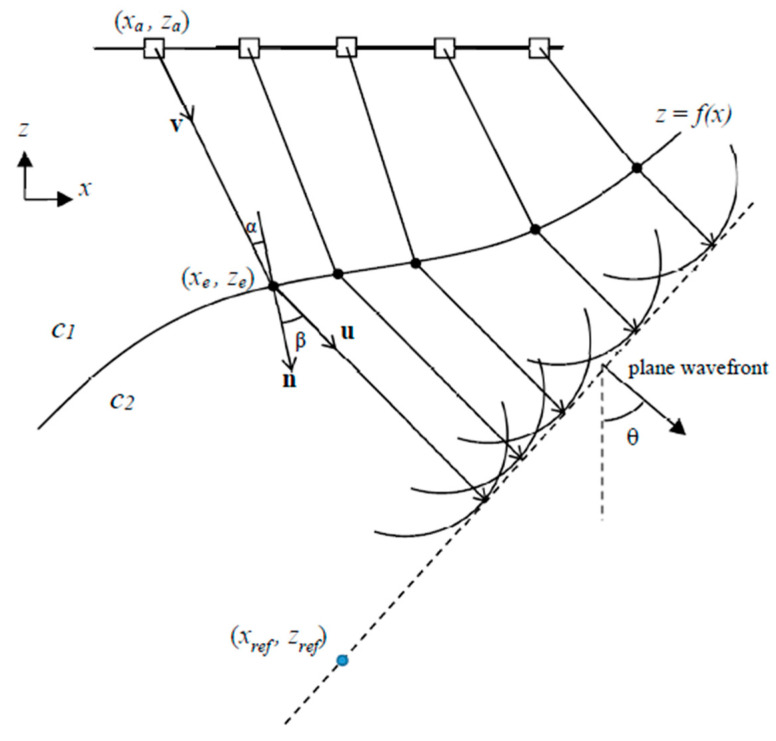
Schematic representation of the geometry for delay law calculation.

The problem to solve is to obtain a set of *N* emission delays *τ_i_* (focal law) that generate a plane wave with propagation direction *θ* beyond an interface (described by *z = f*(*x*)) between two media with propagation velocity *c_1_* and *c_2_* (Figure 1). The plane wave angle *θ* is measured from the array normal to the wavefront normal vectors. With a ray-tracing approach, the problem reduces to find, for each array element (*x_a_, z_a_*), the entry point (*x_e_, z_e_*) at the interface for which the refracted ray into the second medium has an angle *θ* with regard to the *z*-axis. This way, all the refracted rays will be parallel, and the omni-directional waves generated by each array element will assemble a plane-like wavefield with propagation direction *θ* inside the second medium if the emission delays are selected accordingly.

For each array element, the refraction at the interface follows Snell’s Law,

(1) $$\frac{\sin\left(\alpha \right)}{c_{1}}=\frac{\sin\left(\beta \right)}{c_{2}}\;$$

with *α* and *β* representing the incident and refracted angles respectively with regard to the surface normal vector $\vec{n}=\left(n_{x},n_{z}\right)$ at (*x_e_*, *z_e_*). These angles are related with the incident unit vector $\vec{v}=\left(v_{x},v_{z}\right)$ and the refracted unit vector $\vec{u}=\left(u_{x},u_{z}\right)$, both at (*x_e_*, *z_e_*), by

(2) $$\{\begin{array}{c} \sin\left(\alpha \right)=\left|\vec{n}\times \vec{v}\right|=n_{x}v_{z}-n_{z}v_{x}\\ \sin\left(\beta \right)=\left|\vec{n}\times \vec{u}\right|=n_{x}u_{z}-n_{z}u_{x} \end{array}$$

where × is the cross product and | ⋅ | is the modulus operation. The refracted vector $\vec{u}$ is related to the propagation angle *θ* by

(3) $$\vec{u}=\left(u_{x},\;u_{z}\right)=\left(\sin\left(\theta \right),-\cos\left(\theta \right)\right)$$

and the incident vector components are related to the array element and the entry point coordinates (*x_e_*, *z_e_*) by

(4) $$\{\begin{array}{c} v_{x}=\frac{x_{e}-x_{a}}{\sqrt{{\left(x_{e}-x_{a}\right)}^{2}+{\left(z_{e}-z_{a}\right)}^{2}}}\\ v_{z}=\frac{z_{e}-z_{a}}{\sqrt{{\left(x_{e}-x_{a}\right)}^{2}+{\left(z_{e}-z_{a}\right)}^{2}}} \end{array}.$$

If an analytical description of the interface exists, the normal vector $\vec{n}=\left(n_{x},n_{z}\right)$ at (*x_e_*, *z_e_*) pointing into the second medium can be obtained by differentiation

(5) $$\vec{n}=\left(n_{x}\;,\;n_{z}\right)=\left(\frac{df}{dx}\left(x_{e}\right),-1\right)$$

or, alternatively, from the discrete representation $\hat{f}\left(x\right)$ of f(x) according to

(6) $$\vec{n}=\left(\hat{f}\left(x_{e}+\Delta x\right)-\hat{f}\left(x_{e}\right),-\Delta x\right)$$

with Δ*x* the sampling step of the interface. Given that $z_{e}=\hat{f}\left(x_{e}\right)$ and substituting (2), (3), (4), and (6) into (1), the coordinate *x_e_* of the entry point must satisfy the following equation

(7) $$\frac{\hat{f}\left(x_{e}+\Delta x\right)-\hat{f}\left(x_{e}\right)}{\Delta x}\left(\frac{c_{1}}{c_{2}}\frac{\hat{f}(x_{e)}-z_{a}}{\sqrt{{\left(x_{e}-x_{a}\right)}^{2}+{\left(z_{e}-z_{a}\right)}^{2}}}+\cos\left(\theta \right)\right)+\left(\frac{c_{1}}{c_{2}}\frac{x_{e}-x_{a}}{\sqrt{{\left(x_{e}-x_{a}\right)}^{2}+{\left(z_{e}-z_{a}\right)}^{2}}}-\sin\left(\theta \right)\right)=0$$

which can be solved by numerical methods inside the interval $x\in (x_{1},x_{2})$ where the interface is defined.

Once the entry point for each array element is known, the focal law can be obtained from the time-of-flight differences to the plane wavefront at an arbitrary position (reference point from now on) into the second medium. Let $\left(x_{ref},z_{ref}\right)$ be the reference point on the wavefront; then, the time of flight from an array element to the wavefront is

(8) $$tv=\frac{1}{c_{1}}\sqrt{\left({\left(x_{e}-x_{a}\right)}^{2}+{\left(z_{e}-z_{a}\right)}^{2}\right)}+\;+\frac{1}{c_{2}}\left|\left(x_{ref}-x_{e}\;,\;\;z_{ref}-z_{e}\right)\cdot \left(u_{x}\;,u_{z}\right)\right|\;.$$

The reference point can be arbitrarily chosen as long as it satisfies that all entry points are above the wavefront, so it is completely in the second medium.

Finally, the emission focal law is computed by the time-of-flight differences between elements with regard to a common time instant, for example, the maximum TOF:

(9) τ(k)=max(tv)−tv(k).

## 3. Acoustic Field Simulation

**Figure 2:**
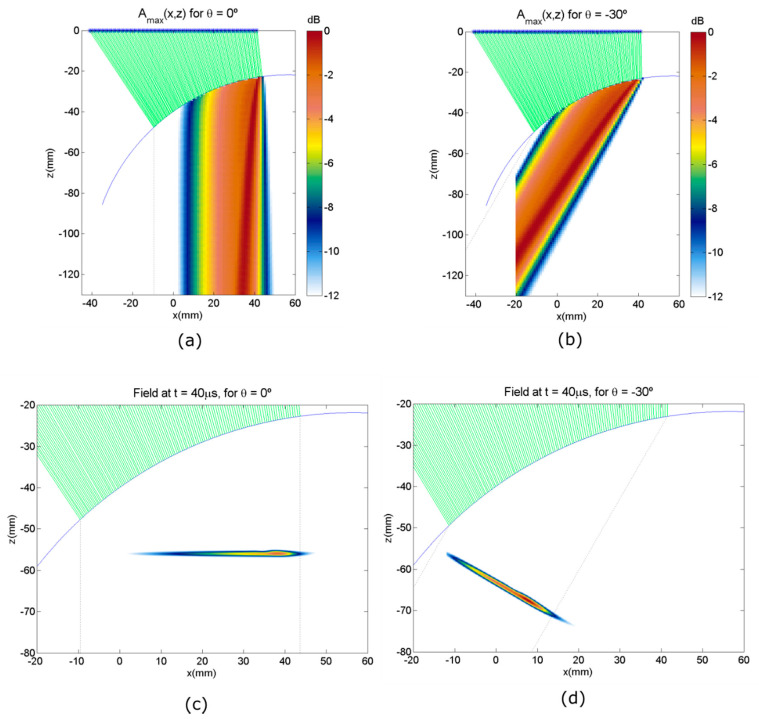
Pulsed wave simulation of a plane wave into a 50 mm radius cylindrical aluminum component. (**a**) Maximum amplitude for *θ* = 0°. (**b**) Maximum amplitude for *θ* = −30°. (**c**) Instantaneous field for *θ* = 0° *t* = 40 μs. (**d**) Instantaneous field for *θ* = −30°, t=40 μs.

Figure 2 shows the pulsed wave simulation with in-house developed software [12] based on the Point Source Model and programmed in Matlab (Matworks Inc., Natick, MA, USA). The time of flight from each array element to each image pixel is obtained by solving for the fastest path through the interface according to the Fermat principle. Then, synthetic signals generated by each element are delayed accordingly and added for computing the field amplitude at each simulation point, accounting for geometric beam spreading, the transmission coefficient between materials, and elements angular directivity. 

The angular sensitivity of an array element can be approximated by [13]

(10) $$A_{\gamma }=\left|sinc\left(\frac{d-g}{\lambda }\sin\left(\gamma \right)\right)\right|$$

with *g* representing the gap between the array elements and *γ* representing the beam propagation angle with regard to the array normal. 

The geometric beam spreading and transmission coefficient are computed as [8]. Let us name $\theta_{i}$ as the incident angle and $L_{1},\;L_{2}$ as the distances traveled by a ray in materials 1 and 2, respectively. Then, the beam spread factor is:

(11) $$A_{bs}=\sqrt{\frac{\beta }{\beta L_{1}+L_{2}}}$$

(12) $$\beta =\frac{{\left(\frac{c_{1}}{c_{2}}\right)}^{2}-\sin^{2}\left(\theta \right)}{\left(\frac{c_{1}}{c_{2}}\right)-\cos^{2}\left(\theta \right)}.$$

Let $\theta_{L}$ be the longitudinal wave (L-wave) refraction angle and $\theta_{S}$ be the shear wave (S-wave) refraction angle. The transmission coefficient for an incident L-wave into a refracted L-wave is (also as [8]):

(13) $$A_{t}=\frac{2c_{1}\rho_{1}}{Nc_{2}\rho_{2}}\cos\left(2\theta_{S}\right)$$

(14) $$N={\left(\frac{c_{3}\;}{c_{2}}\right)}^{2}\sin\left(2\theta_{L}\right)\sin\left(2\theta_{S}\right)+\cos^{2}\left(\theta_{S}\right)+\frac{c_{1}\rho_{1}}{c_{2}\rho_{2}}\frac{\;\cos\left(\theta_{L}\right)}{\;\cos\left(\theta_{i}\right)}$$

where *ρ*_1_,*ρ*_2_ are the densities of materials 1 and 2 respectively, and $c_{3}$ is the S-wave velocity in material 2.

The simulated array is *N* = 128 elements, 5 MHz center frequency, 80% bandwidth, and 0.65 mm pitch, inspecting by water immersion a cylindrical aluminum part with a radius of 50 mm (*c*_1_ = 1.48 mm/us and *c*_2_ = 6.35 mm/us). The emission focal law was obtained from (9) for generating a plane wave with *θ* = 0° and one with *θ* = −30° inside the component.

Figure 2a,b show the maximum amplitude $A_{max}\;\left(x,z\right)$ at each point in the field. Figure 2c,d show the instantaneous field at  t=40 μs. It can be seen that plane waves are generated. Nevertheless, the lateral extent of the plane wave is limited to the region (PWR, Plane Wave Region) between the entry points of the extreme array elements (black dotted lines in Figure 2), and only those pixels inside the PWR should be considered for reception beamforming with this plane wave angle. This is the main limitation of plane wave imaging: The effective imaging area for each emission is limited to the projection of the array aperture at the propagation angle.

Another effect is also shown by the simulation: the field amplitude is not constant across the wavefront, which could lead to echo amplitude differences because of a non-controlled apodization. This behavior is produced mainly by the angular sensitivity of the array elements, the geometric spreading of the beam, and the distribution of the entry points along the interface.

**Figure 3:**
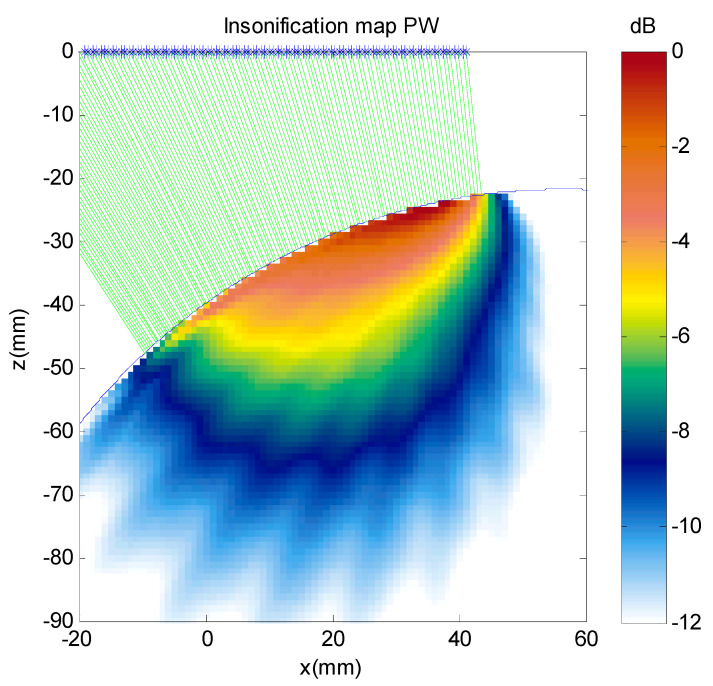
Simulated insonification map for the aluminum cylindrical part for 15 plane waves from −70° to 70° in 10° steps.

A simulation was made for plane waves with angles from −70° to 70° each 10°, to calculate the total insonification map inside the part. This total insonification map represents the average expected acoustic pressure at any point of the part after the emission of a set of plane waves, and it was defined as the summation at each point of the field amplitude (absolute value) generated by each one of the 15 plane waves at that point. Figure 3 shows the insonification map, which is not homogeneous. Depending on the component geometry, this could lead to the presence of “blind zones” with very low insonification or quite large echo amplitude differences for similar reflectors depending on their position in the image. For parts where the geometry is known and fixed during the inspection, at least approximately, it could be useful to simulate the insonification map to detect possible blind regions. It is the case of parts with complex but constant shape by water immersion or curved parts with customized solid wedges. For example, this might help with choosing an optimal position of the transducer relative to the test part for obtaining an homogeneous insonification map.

### 3.1. Simplified Estimation of Amplitude Distribution

In order to develop a better understanding of how the amplitude distribution across a plane front wave is not uniform, a simplified approach was considered to estimate the effect of the non-uniform distribution of entry points on the interface.

The plane wavefront is generated by the interference between approximately cylindrical waves with a center at the entry points (*x_e_*, z_e_), which are not evenly distributed for an arbitrarily shaped interface. The closer the entry points are to each other, the higher the amplitude expected for the plane wave, as more of them will contribute in phase at some point of plane wavefront.

**Figure 4:**
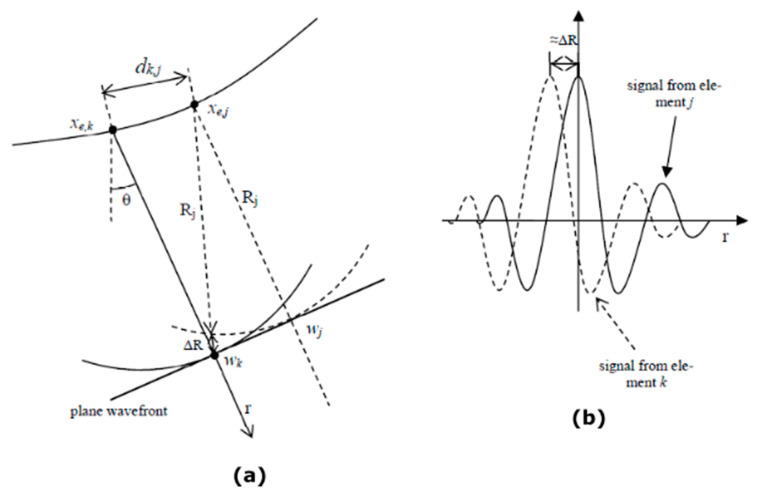
Schematic representation for the estimation of the wavefront amplitude. (**a**) Geometry of the problem and (**b**) Contribution of each element to the plane wavefield.

Figure 4a shows a schematic representation of the problem. The projections of the entry points over the plane wavefront are

(15) $$w_{k}=\left(x_{e,k}+R_{k}\sin\left(\theta \right),\;z_{e,k}-R_{k}\cos\left(\theta \right)\right)$$

with *R_k_* representing the distance from the entry point *k* to the wavefront following the propagation direction *θ*. At point *w_k_*, the wave emitted by the element *j* (dotted trace) will travel behind the plane wave at a distance

(16) $$\Delta R_{k,j}=\sqrt{d_{k,j}^{2}-R_{k}\left(2R_{j}-R_{k}\right)}-R_{j}$$

with *d_k,j_* representing the distance between the entry points of both elements. Figure 4b shows a schematic representation of the individual wavefields along the propagation line *r*, where the wave emitted by the element *j* (dotted) is delayed approximately Δ*R* with regard to the wave emitted by element *k* (solid line), and hence, the interference will not be fully constructive. Furthermore, as *d_k,j_* increases, the contribution of the *jth* element to the wavefront at *w_k_* is reduced, because of the wideband nature of the signals. This way, if the entry points are separated, the amplitude of the resultant plane wave is expected to reduce.

Given the impulse response of the array elements *s(r)*, where *r* is the distance from element to field point, the amplitude of the wavefield at point *w_k_* can be obtained by

(17) $$A_{s}=\sum_{j=1}^{N}s\left(\Delta R_{k,j}\right).$$

For estimating the plane wave amplitude distribution, a simple model for a wideband pulse with frequency *f* and fractional bandwidth *B* is assumed

(18) $$s\left(r\right)=\exp\left(-\frac{r^{2}}{c_{2}b^{2}}\right)\cos\left(\frac{2\pi fr}{c_{2}}\right)$$

with

(19) $$b=\frac{2.355}{2\pi fB}.$$

*f* = 5 MHz and *B* = 0.8 are used in this work. Finally, accounting for the three described effects, the amplitude across the wavefield can be approximated by the product of (10), (11), (13), and (17).

(20) $$A=A_{\gamma }A_{bs}A_{t}A_{s}$$

Solving (17) for all the array elements requires evaluating (17) $NN_{\theta }\;$ times, where $N_{\theta }$ is the number of emitted plane waves. While this is not expected to be a limitation if the interface geometry is constant (the compensation curves are calculated only once), it could be a problem for water immersion inspections of components with non-constant profiles and auto-focusing algorithms. A first-order approximation is to consider as contributing elements to the point *w_k_* only those whose delays Δ*R_k,j_* are below λ/2, and hence, their first positive cycles overlap (constructive interference). Modeling the emitted signal as a square pulse of length *λ*, the expected amplitude at point *w_k_* is just the number of elements that verify the above condition, and hence, (17) simplifies to

(21) $${\tilde{A}}_{s}=\sum_{j=1}^{N}\left|\Delta R_{k,j}\right|<\frac{\lambda }{2}.$$

We define two approximations to Equation (20): ${\tilde{A}}_{1}=A_{\gamma }A_{bs}A_{t}{\tilde{A}}_{s}$ and ${\tilde{A}}_{2}=A_{\gamma }{\tilde{A}}_{s}$. In the second one, we neglect the beam spread and transmission factors.

**Figure 5:**
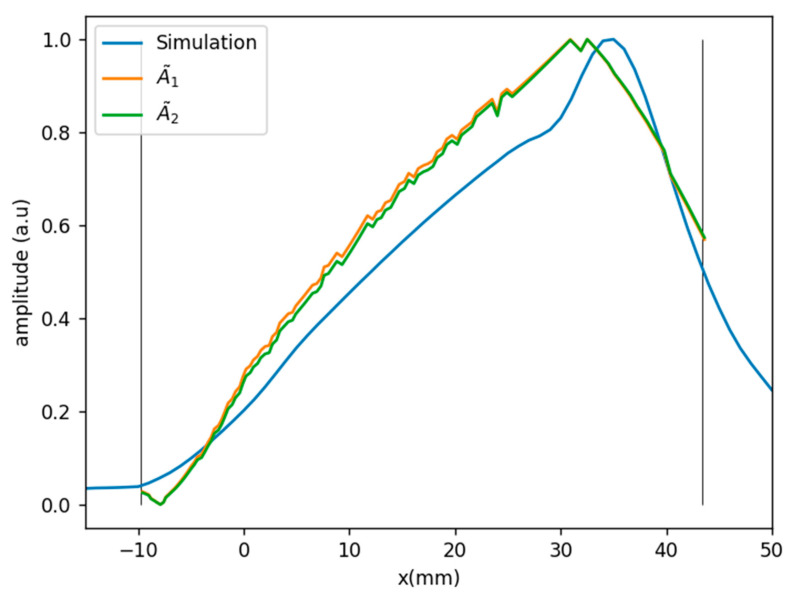
Amplitude distribution along wavefront according to simulation and simplified approximations.

Figure 5 shows the wavefront amplitude according to the simulation and the two approximations ${\tilde{A}}_{1}\;$ and ${\tilde{A}}_{2}\;$, showing that both follow the trend of the simulation and they are almost equal. This suggests that the most relevant factors affecting the amplitude distribution are angular sensitivity and distribution of entry points along the interface. In Section 4.2, we will discuss the feasibility of using this approximation as a compensation factor when computing the PWI image.

### 3.2. Experimental Results

**Figure 6:**
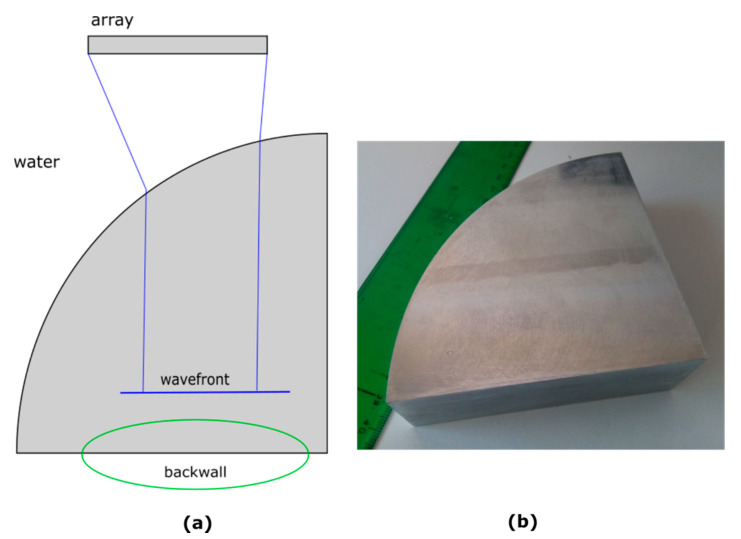
(**a**) Schematic of the experimental setup. (**b**) Test piece.

To test the accuracy of the simulation, an experiment was made with an aluminum specimen in the shape of a 90° circular sector and 100 mm radius (Figure 6b). The specimen was tested in the same conditions as those used in the simulation, with a 5 MHz, 0.65 mm pitch, 128-element array (Imasonic, Voray-sur-l’Ognon, France), and a 128-channel full-parallel phased-array system (Dasel, Madrid, Spain). The interface of the test piece was detected by the pulse-echo method ([9]), and this interface was the one used in the simulation.

The test piece was positioned so that its backwall was parallel to the array, a 0° plane wave was transmitted (Figure 6a), and an image around the backwall was generated. The backwall echo in the image should be (ideally) proportional to the amplitude of the field at *Z* = *L*, where *L* is the distance from the array to the specimen backwall. The amplitude of the backwall echo was evaluated in the image to compare it with the simulation as an indirect way of measuring the field amplitude distribution across the wavefront.

**Figure 7:**
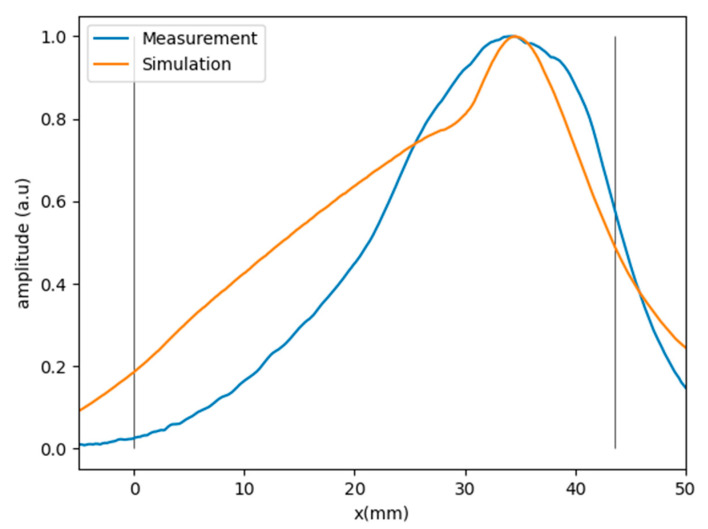
Simulated amplitude on wavefront compared to backwall echoes for each array element.

Figure 7 shows the comparison between simulation and experiment. We can see that that simulation captures the overall shape of the amplitude distribution across the front-wave, but it is not accurate. This inaccuracy is probably associated with the oversimplifications used in the model. In any case, it could be used as a first-order approximation to reduce the PWR extent to avoid summing low-amplitude signals in the reception beamforming process because of low insonification areas. 

## 4. Image Formation Algorithm

### 4.1. Time-of-Flight Calculation

After each plane wave emission, the signals received by all the array elements are used to generate a whole image. Repeating the process with different plane wave angles and averaging those single images, a higher quality one is obtained in a similar way as in the Total Focusing Method (TFM). The main difference with regard to TFM is the emission time-of-flight calculation from the array to the pixel, which in PWI depends on the plane wave angle *θ*. 

**Figure 8:**
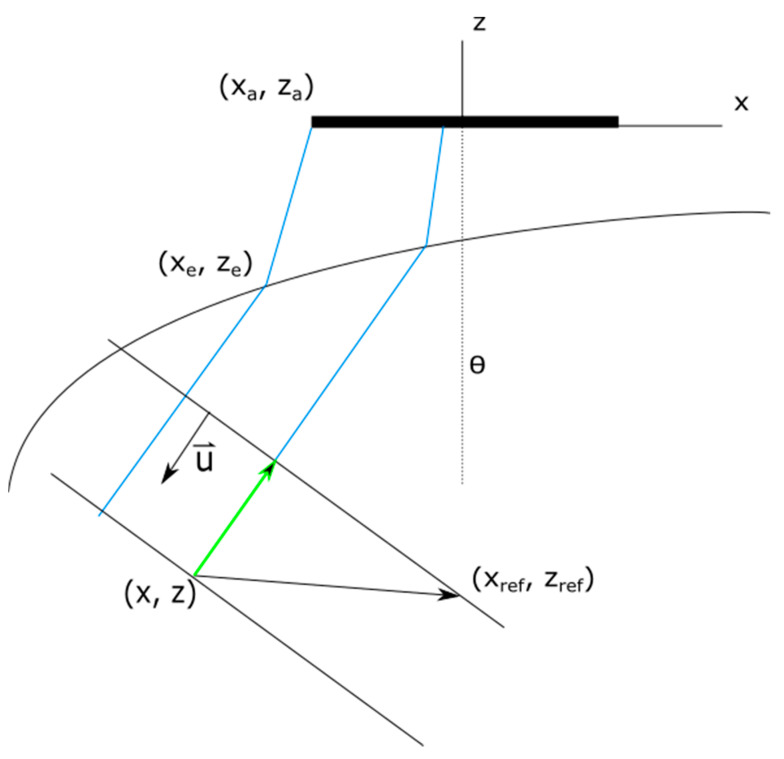
Sketch showing the geometry of refraction and the variables used for time-of-flight calculation.

The time-of-flight $t_{pw}=(\theta ,x,z)$ to a point (x,z) (Figure 8) can be calculated using the reference point used in Equation (8):

(22) $$t_{ref}=t_{PW}\left(\theta ,\;x_{ref},\;z_{ref}\right)=\max\left(tv\right).$$

Then,

(23) $$t_{PW}\left(\theta ,x,z\right)=t_{ref}-\vec{u}\cdot \left(\left(x_{ref},z_{ref}\right)-\left(x,z\right)\right).$$

The dot • means the scalar product.

For the reception TOF from the pixel (*x*,*z*) to the receiving element *k*, the Fermat principle is applied as in TFM. The $x_{o}(k)$ coordinate of the reception exit point is obtained by minimizing the TOF function

(24) $$t_{TFM}\left(x,z\right)=\min\left(\frac{1}{c_{1}}\sqrt{{\left(x_{o}-x_{a}\right)}^{2}+{\left(f(x_{o})-z_{a}\right)}^{2}}+\frac{1}{c_{2}}\sqrt{{\left(x-x_{o}\right)}^{2}+{\left(z-f(x_{o})\right)}^{2}}\right).$$

In this case, the reception TOF does not depend on the angle *θ*, as the backscattered wave is considered omni-directional. Therefore, the reception TOF is the same for all the plane wave angles.

Finally, the round-trip TOF for pixel and element *k* to be used for indexing the received A-scans during beamforming is

(25) $$t\left(\theta ,x,z,k\right)=t_{PW}\left(\theta ,x,z\right)+t_{TFM}\left(x,z,k\right).$$

### 4.2. Spatial Weighting

According to the results of Section 2, the wavefront can be considered flat only between the projection of the entry points of the extreme array elements *e_1_* and *e_k_* along the propagation direction *θ.* Additionally, the field amplitude is not uniform inside the PWR.

Therefore, for a pixel (x,z)*,* only those *θ* where it lies inside the PWR will be considered. Thus, the equation to calculate the pixel intensity I(x,z) is

(26) $$I\left(x,z\right)=\frac{\sum_{\theta \;}\sum_{k}w\left(\theta ,x,z\right)s\left(t\left(x,z,k\right),k\right)}{\sum_{\theta }w\left(\theta ,x,z\right)}$$

where *w*(*θ*,*x*,*z*) = 0 outside the PWR. Inside the PWR, we propose two strategies: constant weighting (CW) and simulation-based weighting (SBW).

In the case of constant weighting:

(27) $$w_{PWR}\left(\theta ,x,z\right)=\{\begin{array}{c} 1\;\;\left(x,z\right)\in PWR\\ 0\;\;\left(x,z\right)\notin PWR \end{array}.$$

For the simulation-based case, we try to compensate the non-uniform amplitude distribution using the results obtained in Section 2. Two options were tested: SBW1 uses the simulated acoustic field ψ(θ, x, z), and SBW2 uses the approximation ${\tilde{A}}_{2}$, which were computed at a wavefront in the middle of the image.

(28) $$w_{SBW1}\left(\theta ,x,z\right)=\frac{1}{\psi \left(\theta ,x,z\right)}\;\;\;\;$$

(29) $$w_{SBW2}\left(\theta ,x,z\right)=\{\begin{array}{c} \frac{1}{{\tilde{A}}_{2}\left(\theta ,\;x,\;z\right)}\;\left(x,z\right)\in PWR\\ 0\;\;\;\;\;\;\;\;\;\;\;\;\;\;\left(x,z\right)\notin PWR \end{array}$$

Another strategy tested was to compute the image using CW and multiplying the result by the insonification field. We will call this strategy insonification field weighting (IFW), and we have also two options:

(30) $$I_{IFW1}\left(x,z\right)=\frac{I_{CW}\left(x,z\right)}{\sum_{\theta }w_{SBW1\left(\theta ,x,z\right)}}$$

(31) $$I_{IFW2}\left(x,z\right)=\frac{I_{CW}\left(x,z\right)}{\sum_{\theta }w_{SBW2\left(\theta ,x,z\right)}}.$$

## 5. Materials and Methods

**Figure 9:**
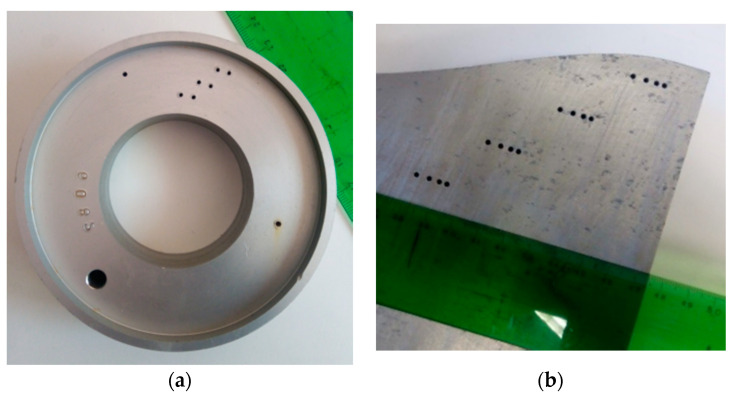
(**a**) Test piece A. (**b**) Test piece B.

Two aluminum test pieces were used to test the image formation algorithms. Test piece A (Figure 9a) has a circular shape and two parallel rows of holes. Test piece B (Figure 9b) has a curved interface with concave and convex sectors, and it has multiple rows of holes. Both were tested in water immersion with an array with *N =* 128 elements, 5 MHz center frequency, 80% bandwidth, and 0.65 mm pitch (Imasonic, France). A full parallel 128-channel phased array system was used for excitation and acquisition (Dasel, Spain).

The pitch-catch method [9] was used to detect the interface, which was used to compute focal laws according to Equation (9). The detected interface was also used to simulate the acoustic field for the simulation-based weighting. PWI acquisitions were taken with −70° < *θ* < 70° every 10° for a total of 15 emissions.

## 6. Results and Discussion

### 6.1. Partial Images

**Figure 10:**
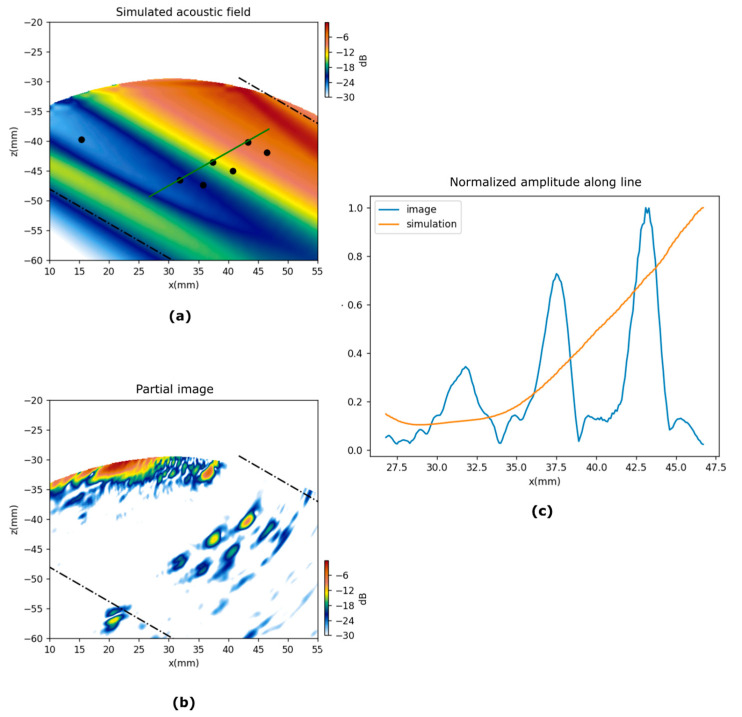
Simulation and partial PWI images for emission at 60°, test piece A. (**a**) Simulated acoustic field, the dots represent the holes. (**b**) Partial PWI image. (**c**) Amplitude along the green line in (**a**).

**Figure 11:**
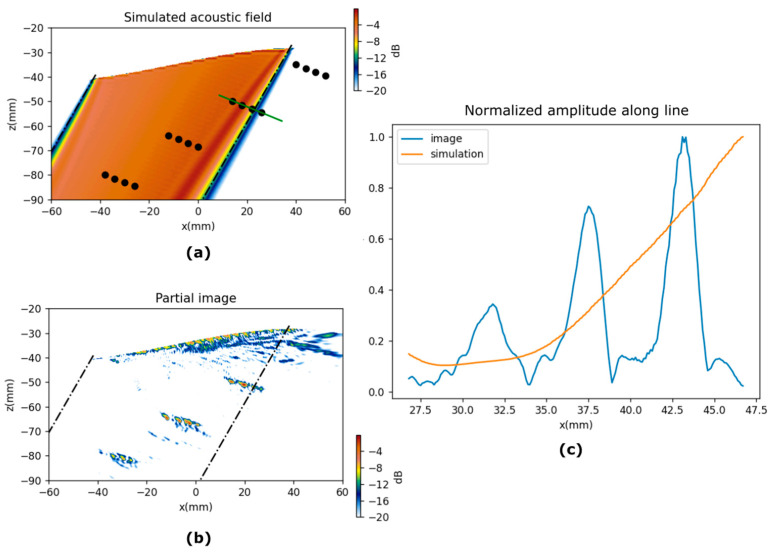
Simulation and partial PWI images for emission at −30°, test piece B. (**a**) Simulated acoustic field, the dots represent the holes. (**b**) Partial PWI image. (**c**) Amplitude along green line in (**a**).

The PWI images are the coherent summation of the partial images computed for each emission. Figure 10 and Figure 11 show partial images at 60° and −30° for test pieces A and B respectively, and the corresponding simulated fields. In both figures, an amplitude profile is plotted along a line that goes through a group of holes. Both figures show how the amplitude of the indications follows the trend of the simulated acoustic field. We can see clearly in these figures the effect of the non-uniform amplitude on the acoustic field across a plane wavefront.

It is worth mentioning the low-amplitude region in specimen A and 60° emission. Different than expected, a non-monotonous pattern is observed along the wavefront, which reduces the echo amplitude of three defects that are not located at the edge of the PWR. This counterintuitive behavior should be accounted for in analysis when designing plane wave inspections in complex-shaped parts.

### 6.2. Full Images

**Figure 12:**
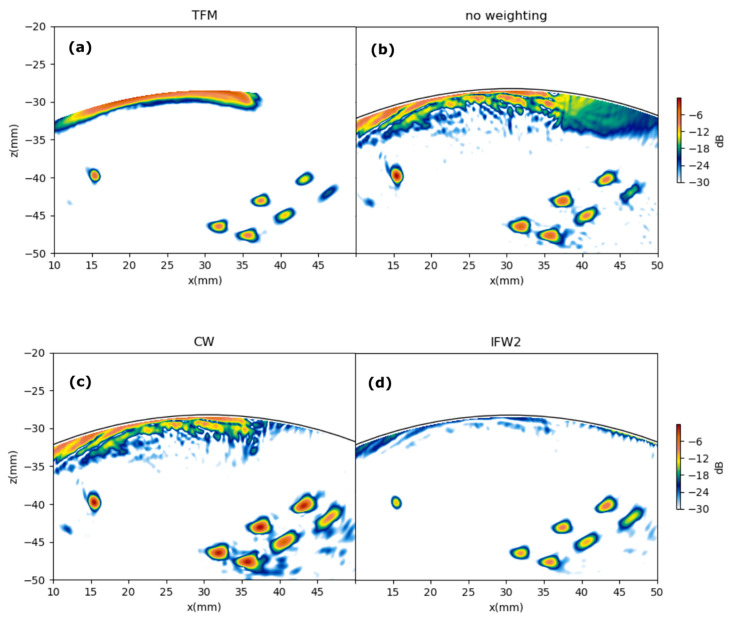
TFM and PWI images of test piece A. (**a**) TFM. (**b**) PWI with no weighting. (**c**) PWI with CW. (**d**) PWI with IFW2 (Insonification Field Weighting defined in Equation (31)). Color scale span is −30 dB.

**Figure 13:**
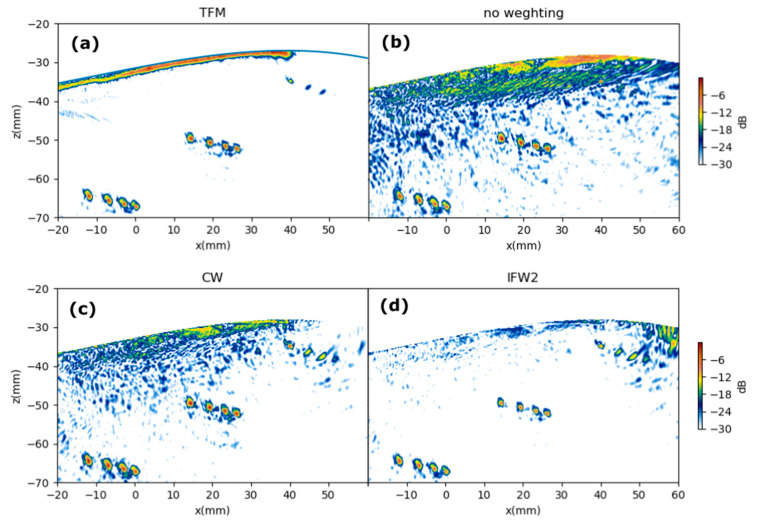
TFM and PWI images of test piece B. (**a**) TFM. (**b**) PWI with no weighting. (**c**) PWI with CW. (**d**) PWI with IFW2. Color scale span is −30 dB.

Figure 12 and Figure 13 show the resulting images for specimens A and B, respectively. Four images are shown in each case: TFM, PWI with no weighting (*w*(*θ*,*x*,*z*) = 1 for all points in the image), PWI with CW, and PWI with SBW1. For the TFM images, 128 firings were made, and for the PWI images, 15 firings were made.

Figure 12 and Figure 13 show a contrast improvement in the PWI images when applying the proposed pixel weighting with regard to no-weighting (NW). The background noise generated by the low-level amplitude signals outside the PWR is reduced when weighting, which is clearly seen in the top-right holes region of Figure 13c,d when compared with that in Figure 13b. 

A first result observed in Figure 11 is that both the CW and IFW2 strategies produce similar echo amplitudes for the seven holes in test piece A. This is important in NDT because the three holes are the same size, so they should ideally produce equal intensity indications in the image. 

In Figure 11, the contrast enhancement of both weighting schemes relative to no weighting is more evident. Both weighting schemes perform also better than TFM, in which no spatial weighting is applied. 

The equalization of equal defects amplitudes obtained with the weighting scheme was quantified by measuring the peak value of each indication and computing, for each image, the standard deviation of these peak values as a fraction of the maximum peak value. Results are shown in Table 1. It is seen that PWI with IFW2 has the best performance in both test pieces. 

### 6.3. Fringing Artifacts

**Figure 14:**
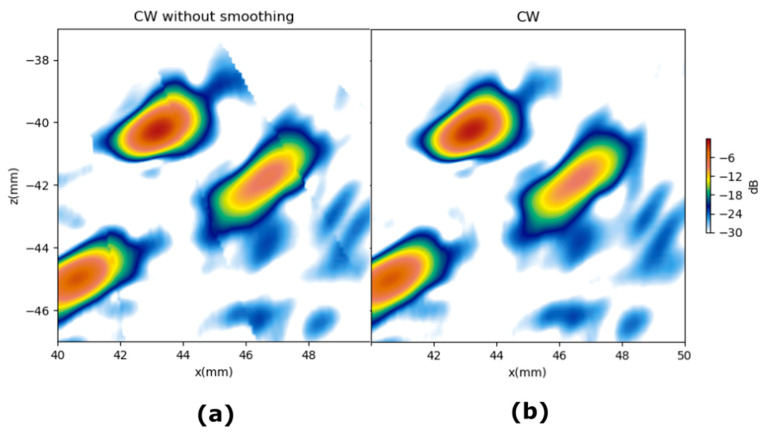
Fringing artifact. (**a**) Discontinuous weighting mask. (**b**) Smoothed weighting mask.

The weighting schemes CW, SBW2, and IFW2 use masks with a discontinuity at the edge of the PWR. This produces a fringing artifact in the PWI image, such as the one shown in Figure 14a. This artefact can be smoothed out by applying a filter to the image, but this has the disadvantage of lowering the lateral resolution. To avoid this, we propose applying a smoothing filter to the weighting mask *w.* Figure 13b shows how the use of a smoothed mask erases the fringing artefact. The filter used was a 9 × 9 pixels averaging filter.

## 7. Conclusions

We have shown through simulation how plane waves emitted inside a component with refraction on its surface could lead to non-uniform insonification. This phenomenon could potentially produce unexpected amplitude variations for equivalent size and shape defects, which is a non-desired effect in NDT. Furthermore, “blind zones” could be produced if the insonification amplitude heavily drops in some region inside the image.

An experiment was done to measure the front wave amplitude distribution using an aluminum specimen in the shape of a 90° circular sector and 100 mm radius. The specimen is representative of the frequent cylindrical geometry found in many real pieces. Using a plane wavefront parallel to the backwall, a TFM image was done to estimate the amplitude distribution, showing a non-homogenous amplitude distribution, which is in agreement with the simulation results.

It was shown that even a simple simulation algorithm can predict the behavior of the generated plane waves, and a pixel weighting procedure was proposed to reduce the impact of the insonification inhomogeneity in the echoes amplitude. This method was experimentally validated with two components with non-planar surfaces. 

The weighting strategies tested show significant improvement with respect to the no weighting case. In addition, from all the weighting strategies tested, the IFW2 provided better equalization of the indication amplitudes of equal holes in different positions of both test pieces. 

It is expected that a more accurate simulation will improve the performance of SBW1 and IFW1 in complex scenarios, but with the cost of a larger computation time. This could be prohibitive for real-time varying shape components inspection, but it could be feasible for solid wedges or relatively constant shape immersion tests where the simulation can be performed only once. On the other hand, the approximation given by (21), which is the one used for IFW2, might be computed significantly faster and so could be a candidate for real-time implementation in high-speed auto-focused systems.

## Figures and Tables

**Table 1:** Standard deviation of indications peak value (fraction of maximum peak value).

	Test Piece A	Test Piece B
TFM	0.24	0.31
PWI-no weighting	0.23	0.27
PWI-W	0.19	0.27
PWI-SBW1	0.23	0.20
PWI-SBW2	0.17	0.25
PWI-IFW1	0.17	0.24
PWI-IFW2	0.15	0.20
